# How does childhood socioeconomic position affect overweight and obesity in adolescence and early adulthood: a longitudinal study

**DOI:** 10.1186/s40608-018-0210-8

**Published:** 2018-12-03

**Authors:** Per Hoegh Poulsen, Karin Biering, Trine Nøhr Winding, Ellen Aagaard Nohr, Johan Hviid Andersen

**Affiliations:** 1 Danish Ramazzini Centre, Department of Occupational Medicine, University Research Clinic, Regional Hospital West Jutland, Gl. Landevej 61, 7400 Herning, Denmark; 2 Institute of Clinical Research, Department of Obstetrics & Gynecology, Odense University Hospital, University of Southern Denmark, Odense, Denmark

**Keywords:** Young people, Childhood socioeconomic exposures, Timing of exposure, Overweight, Gender-specific differences

## Abstract

**Background:**

Childhood socioeconomic position (SEP) has previously been associated with increased risk of overweight among children and adolescents. However, it remains uncertain whether the timing of exposure is important in relation to developing overweight in early adulthood. We aimed to examine how SEP during early (0–8 years) and late childhood (9–14 years) relates to overweight at age 15, 18 and 21.

**Methods:**

Longitudinal study in Western Denmark of 2879 young people (aged 15 in 2004). Exposure variables from registers were yearly household income, parental highest educational level and parental labour market participation (LMP), supplemented with questionnaire information about “family functioning” (age 15). Outcome variables were overweight and obesity, measured at three-time points.

We analyzed the adjusted associations between childhood SEP and overweight and obesity using multinomial logistic regression, stratified on gender.

**Results:**

**Early childhood:** Parental lower educational level increased girls’ risk of overweight and obesity at age 18 and 21 between RR = 1.8 (95% CI 1.0;3.4) and RR = 5.2 (95% CI 1.4;19.3). Girls reporting poor “family functioning” had up to twice the risk of overweight and obesity at age 21. Boys, whose fathers had a lower level of education had up to 2.4 times the risk of obesity at age 21. Parental low LMP increased boys’ risk of obesity at age 18 and 21 between RR = 2.2 (95% CI 1.3;3.8) and RR = 2.8 (95% CI 1.3;6.1). **Late childhood:** Parental lower level of education tripled the risk of overweight and obesity among girls at age 18 and among both genders at age 21.

**Conclusion:**

This study confirmed to some extent that economic, social and psychological insecurity and inequality as measured by lower parental educational level, lower household income, low labour market participation and poor family function during childhood was associated with an increased risk of overweight and especially obesity in adolescence and early adulthood in both genders. Despite some imprecise measures, the direction of the associations pointed to several associations, which all were in the hypothesized direction. Timing of lower household income and parental low LMP in childhood seemed to be gender-specific in some way, but this warrants more studies.

**Electronic supplementary material:**

The online version of this article (10.1186/s40608-018-0210-8) contains supplementary material, which is available to authorized users.

## Background

Prevalence of overweight and obesity has increased worldwide over the last decades [1]. In Denmark, the overall prevalence of overweight and obese children and adolescents appears to be stable or slowly declining [2]. Recent findings show that 26% of young Danes aged 16–24 are overweight and obese. This percentage has been increasing among both genders since 2010, with the highest prevalence among those having primary school as the highest level of education [3].

Overweight and obesity has traditionally been associated with a thermodynamic explanatory model [4] combined with genetics, where preventive initiatives primarily have focused on healthy diet, increased physical activity and lifestyle changes, showing modest associations [5]. In recent years theories about economic, social and psychological insecurity and inequality in relation to obesity has gained ground [6, 7]. The theory which involves social insecurity pursues the hypothesis that obesity could be a healthy active response to an expected future lack of energy [8]. In higher income countries with easily access to energy-dense food, exposure to economic, social and psychological insecurity and inequality in terms of low socioeconomic position (SEP) may induce excessive weight gain [9, 10].

According to SEP and future physical health, Newton et al. concluded that the inverse relationship between low life-course SEP and obesity was consistent for women, not for men [11]. These findings were also reported by a recent review, which concluded that perceived financial hardship before the age of 16 and having an unemployed father were associated with a higher Body Mass Index (BMI) in males. Among females, it was primarily low paternal education level which was associated with a higher BMI [12]. Brisbois et al. found that father’s lower employment status as a proxy for childhood SEP appeared to be an early (before the age of 5) marker of obesity among adults in both genders [13].

The health of young people is strongly affected by social factors at a personal, family and societal level. One of the strongest determinants of health is income inequality [14]. In the review by Halliday et al. it was argued that the social factor “family functioning” may be an important risk factor for physical health, hence poor “family functioning” was associated with an increased risk of overweight and obesity among children and adolescents [15]. Family functioning covers a person’s perception on e.g. how crisis may be dealt with in the nearest family, thereby adding an individual perspective to the family level.

Previous research has predominantly focused on early childhood in relation to physical health in later childhood and adolescence. However, research using longitudinal datasets to address and explore the pathways and mechanisms by which low income/SEP exerts its long term effect on physical health are needed [16]. Especially the age-period 18–26 years appears to be critical by having profound and long-lasting implications for young people’s future health and well-being [17]. Another sensitive period of development appears to be adolescence thereby indicating that the timing of SEP exposure may be an important issue to address in relation to future health problems [18].

How does the timing of several socioeconomic exposures at the family level during the entire span of childhood relate to later risk of overweight and obesity? By including both objective and subjective exposure variables in a longitudinal design we aimed to contribute to the scientific knowledge in this field. Our purpose was to explore the association between SEP during early childhood (0–8 year) and late childhood (9–14 year) and overweight and obesity at age 15, 18 and 21 years.

## Methods

### Design and population

The study was a prospective cohort study. Data was collected as part of the West Jutland Cohort Study (VestLiv), which is an ongoing Danish longitudinal study following a complete cohort of young people born in 1989 and residing in the former Ringkoebing County in 2004. The source population comprised 3681 young people. Recruitment of participants took place at the schools within the county where a baseline questionnaire was completed during school hours in 2004 when the participants were approximately 15 years old. Those not at school on the day of collection received the questionnaire by mail. Of the potential 3681 responders, 3054 (83%) participated in this study. All the potential responders in 2004 were re-invited to participate at the latter waves.

The project has so far included waves of questionnaires in 2004 (age 15), 2007 (age 18) and 2010 (age 21) (http://www.vestliv.dk), which furthermore have been supplemented with a range of register-based information.

A more thorough information on recruitment and data collection has been presented elsewhere [19].

**Fig. 1:**
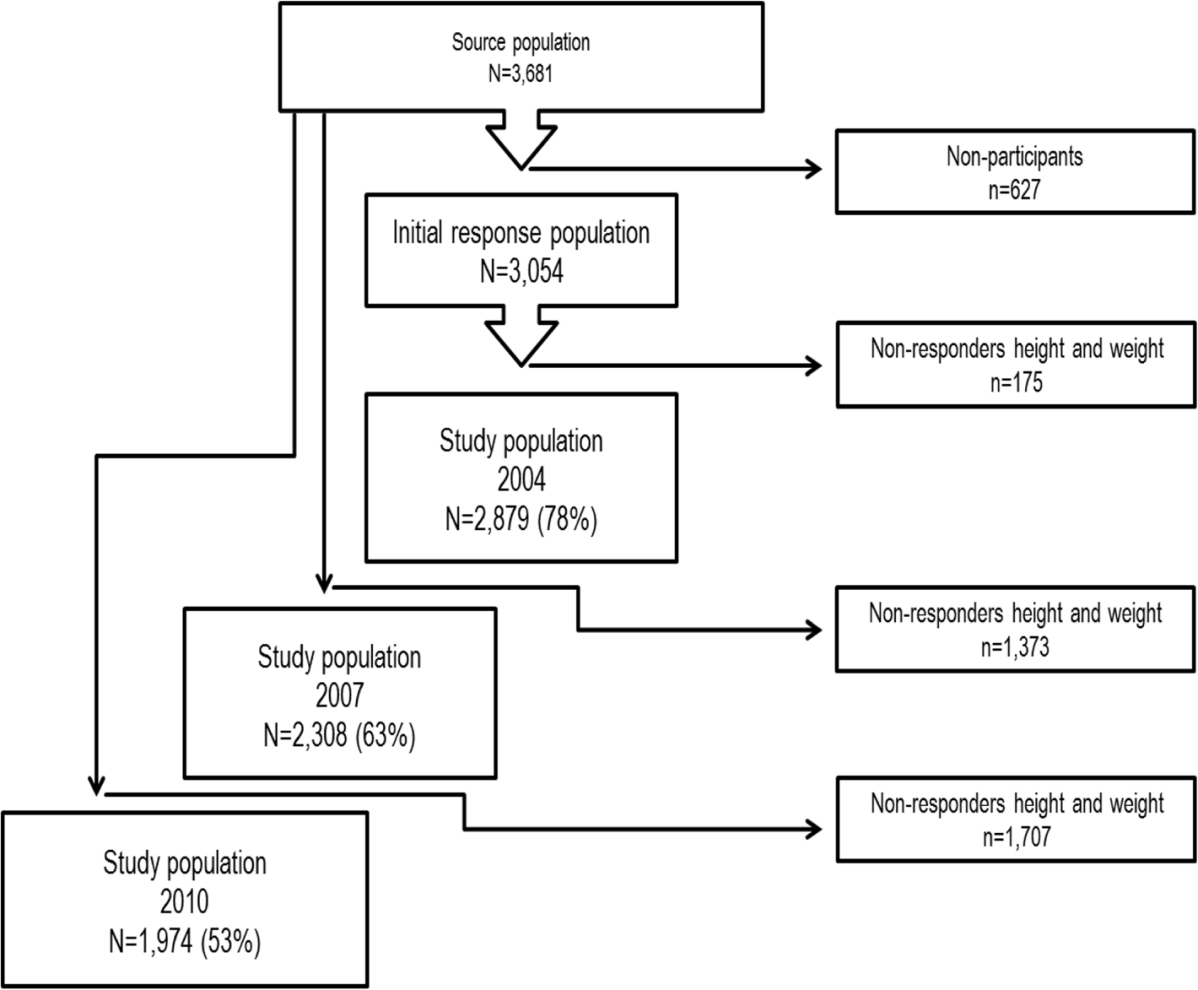
Baseline participation and participation to follow-ups in 2007 and 2010

Participants were included in this current study if they had responded to the questions about weight and height in one of the three questionnaire rounds. This was obtained for 2879 in 2004, 2308 in 2007 and 1974 in 2010. Attrition and missing data reduced the sample as shown in Fig. 1.

Data comprised a combination of both questionnaire data and register data. In Denmark, every citizen is provided with a CPR-number (Civil Registration Number) at birth (or upon entry for immigrants), which allowed the researchers to link the CPR number of each child to parental information from registers [20].

### Definition of outcome

The outcome measure was overweight and obesity, defined by Body Mass Index (BMI), which was calculated from self-reported weight and height (weight/height^2^) collected at all three questionnaire rounds (age 15, 18 and 21).

At 18 and 21 years of age, participants were categorized as “normal weight” (BMI ≤24.99), “overweight” (BMI 25 to 27.49) or “obese” (BMI ≥27.50), due to additional cut-off points from the Global Database on Body Mass Index [21]. Because there were very few 15 year old obese in this cohort, participants were at this age dichotomized into “normal weight” (< 23.29 kg/m^2^ for boys and < 23.94 kg/m^2^ for girls), and “overweight” (BMI ≥23.29 kg/m^2^ for boys and BMI ≥23.94 kg/m^2^ for girls) using thresholds for 15 year old girls and boys [22].

### Definition of exposures

Childhood SEP was defined by yearly household income, parental highest educational level, parental labour market participation (LMP) and “family functioning” to uncover aspects of both economic and social inequality at the family level. Two age-intervals in childhood was applied; early childhood (0–8 year) and late childhood (9–14 year).

Information on yearly household income was from the Danish register on personal income and transfer payments [23] and parental highest educational level was derived from different educational registers [24]. Parental LMP was derived from the DREAM register [25], which provides information on social benefits and information on payments related to e.g. unemployment benefits and sickness absence compensation on a weekly basis [25].

Yearly household income was a continuous variable collected each year from 1989 (birth of child) and onwards until 2003 (age 14). The variable consisted of information on all residents above 18 years in the household living together with the child. Information about household income had to be available for at least five years in an age-interval in childhood to calculate a mean value. Household income was categorized into low, medium or high income according to the 33.3rd and 66.6th percentile.

Information about parental highest educational level was included for each parent from the year 1989 and year 2003 and was divided into three categories: < 10, 10–13, > 13 years of education. If information on highest educational level was missing for year 2003, information was used from previous years (last observation carried forward).

Parental LMP was defined according to the degree of receiving social benefits within each year from 1991 to 2003. When we defined this variable, we omitted payments due to receiving maternity leave benefits or state educational grants. Information about LMP had to be available for at least four years in an age-interval in childhood to calculate a mean LMP score [26]. LMP was a continuous variable in the range from 0 to 100 and calculated as a yearly mean LMP score for each parent in each age-interval and dichotomized into “high LMP” and “low LMP” at a cut off value of ≥0.80 indicating high LMP [27].

Information about “family functioning” was obtained from the initial questionnaire and is a categorical variable with 12 items based on the General functioning subscale of the McMaster Family Assessment Device (FAD), developed by Epstein et al. This variable assesses the overall health/pathology of the family. We calculated a mean value for the 12 items and dichotomized the variable at the 75th percentile of the mean value indicating poor “family functioning” at ≥2.08 [28].

#### Additional variables

High birth-weight and parental marital status have previously been associated with an increased risk of later overweight and obesity [29–32].

Birth-weight was obtained from the Danish Medical Birth Register; a national register which contains information about all births in hospital and home births [33]. We applied the variable split home as a dichotomous variable with yearly information obtained from the CPR register on whether the child lived together with both parents or not. In this study, split home in 1989 (at birth) and split home in 2003 (age 14) was applied for early and late childhood, in that order.

### Statistical methods

Multinomial logistic regression models were used to calculate the associations between the exposure variables during early or late childhood and overweight and obesity at age 18 and 21. Results are presented with unadjusted and adjusted relative risks (ARR). The adjusted results are shown with 95% confidence interval (95% CI). At age 15, we applied logistic regression models to calculate the associations between the exposure variables and overweight. Correlation analyses were carried out for all the exposure variables by Spearman’s rank correlation coefficient. The correlations between mean yearly household income in early and late childhood and between parental highest educational level in early and late childhood was very high. The correlation between mean yearly household income and mean LMP of the father showed a Spearman’s rho = 0.3631. The correlation coefficient between mean LMP of the mother and mother’s highest educational level in 1989 was rho = 0.2925 (Additional file 1: Table S1). Since the rest of the correlation coefficients were similar or lower, all analyses were mutually adjusted for all other exposure variables and birth weight as a continuous variable. Furthermore, the variable split home 1989 was applied to early childhood adjustments, whereas the variable split home 2003 was applied to late childhood adjustments. Moreover, when examining the associations between mean yearly household income in late childhood and overweight and obesity in adolescence and early adulthood, we also adjusted the associations for the yearly household income exposure in early childhood. When we did the analyses for parental LMP in late childhood and overweight and obesity, we also adjusted the associations for the same exposure variable in early childhood. These adjustments were applied to take the effect of the early childhood exposure into account.

Sub-analyses explored whether non-participants at baseline were significantly different from participants with respect to SEP (tables not shown). Data-analysis was performed by the statistical package Stata, statistical software version 14.2 (Stata Corporation, College Station, Texas, USA).

### Ethics

Use of the data is carried out under the same conditions and with the same purpose as when originally collected and based on approval from the Danish Data Protection Agency and their rules of data protection. According to Danish law, approval by the Ethics Committee and written informed consent was not required in questionnaire-based and register-based project.

## Results

Descriptive data of the exposure variables in relation to the outcome at age 18 and 21 are presented for each gender in Table 1.

**Table 1:** Distribution of exposure variables in relation to normal weight, overweight and obesity at age 18 and 21 (*N* = 2308)

Variables	Girls								Boys							
	18				21				18				21			
		Normal	Overweight	Obesity		Normal	Overweight	Obesity		Normal	Overweight	Obesity		Normal	Overweight	Obesity
Early childhood	n	n (%)	n (%)	n (%)	n	n (%)	n (%)	n (%)	n	n (%)	n (%)	n (%)	n	n (%)	n (%)	n (%)
Income^a^	1200				1060				1030				861			
High		392 (88)	30 (7)	22 (5)		291 (78)	41 (11)	40 (11)		319 (82)	42 (11)	27 (7)		241 (74)	57 (17)	30 (9)
Medium		354 (83)	42 (10)	29 (7)		274 (72)	46 (12)	61 (16)		310 (83)	33 (9)	29 (8)		224 (72)	47 (15)	42 (13)
Low		284 (86)	29 (9)	18 (5)		227 (74)	33 (11)	47 (15)		209 (77)	29 (11)	32 (12)		156 (71)	30 (14)	34 (15)
LMP^b^ (mother)	1239				1085				1066				883			
High score		756 (88)	61 (7)	47 (5)		590 (78)	71 (9)	99 (13)		637 (82)	77 (10)	58 (8)		490 (77)	86 (13)	62 (10)
Low score		312 (83)	40 (11)	23 (6)		228 (70)	48 (15)	49 (15)		231 (78)	32 (11)	31 (11)		152 (62)	48 (20)	45 (18)
LMP^b^ (father)	1228				1074				1062				881			
High score		928 (86)	85 (8)	63 (6)		715 (75)	105 (11)	130 (14)		778 (82)	93 (10)	73 (8)		576 (73)	124 (15)	93 (12)
Low score		131 (86)	15 (10)	6 (4)		94 (76)	14 (11)	16 (13)		87 (74)	15 (13)	16 (13)		64 (73)	10 (11)	14 (16)
Highest educational level (mother) 1989	1165				1034				1001				841			
1 (> 13 yr)		242 (90)	17 (6)	10 (4)		197 (83)	17 (7)	23 (10)		228 (86)	22 (8)	17 (6)		191 (80)	27 (11)	21 (9)
2 (10–13 yr)		480 (88)	42 (8)	23 (4)		390 (79)	47 (9)	59 (12)		347 (83)	46 (11)	27 (6)		243 (72)	62 (18)	34 (10)
3 (< 10 yr)		279 (79)	38 (11)	34 (10)		187 (62)	52 (17)	62 (21)		238 (76)	34 (11)	42 (13)		174 (66)	43 (16)	46 (18)
Highest educational level (father) 1989	1119				984				965				801			
1 (> 13 yr)		225 (92)	15 (6)	4 (2)		177 (85)	12 (6)	19 (9)		189 (84)	20 (9)	15 (7)		163 (81)	25 (12)	13 (7)
2 (10–13 yr)		493 (84)	56 (9)	39 (7)		375 (73)	59 (12)	78 (15)		401 (82)	49 (10)	38 (8)		274 (71)	63 (17)	47 (12)
3 (< 10 yr)		241 (84)	25 (9)	21 (7)		180 (68)	41 (16)	43 (16)		195 (77)	26 (10)	32 (13)		142 (66)	40 (18)	34 (16)
Late childhood																
Income^a^	1222				1072				1053				876			
High		408 (88)	34 (8)	20 (4)		303 (78)	46 (12)	39 (10)		325 (83)	42 (11)	26 (6)		238 (72)	60 (18)	33 (10)
Medium		365 (84)	36 (8)	32 (8)		293 (75)	37 (10)	60 (15)		308 (82)	33 (9)	34 (9)		229 (73)	48 (15)	39 (12)
Low		277 (85)	32 (10)	18 (5)		210 (71)	36 (12)	49 (17)		225 (79)	31 (11)	29 (10)		170 (74)	26 (11)	33 (15)
LMP^b^ (mother)	1228				1076				1059				879			
High score		827 (88)	67 (7)	47 (5)		633 (77)	83 (10)	106 (13)		685 (82)	84 (10)	68 (8)		511 (73)	107 (15)	83 (12)
Low score		230 (80)	34 (12)	23 (8)		177 (69)	36 (14)	42 (17)		178 (80)	23 (10)	21 (10)		129 (72)	25 (14)	24 (14)
LMP^b^ (father)	1213				1057				1049				868			
High score		921 (87)	87 (8)	56 (5)		718 (76)	100 (11)	119 (13)		777 (82)	99 (10)	75 (8)		564 (71)	130 (17)	95 (12)
Low score		126 (84)	13 (9)	10 (7)		80 (67)	18 (15)	22 (18)		77 (79)	9 (9)	12 (12)		66 (83)	3 (4)	10 (13)
Highest educational level (mother) 2003	1225				1076				1048				876			
1 (> 13 yr)		338 (91)	22 (6)	13 (3)		269 (84)	24 (7)	29 (9)		296 (84)	32 (9)	24 (7)		241 (79)	38 (12)	27 (9)
2 (10–13 yr)		494 (87)	48 (8)	28 (5)		392 (76)	55 (11)	70 (13)		378 (82)	53 (12)	29 (6)		258 (71)	64 (17)	43 (12)
3 (< 10 yr)		224 (79)	30 (11)	28 (10)		152 (64)	39 (17)	46 (19)		181 (77)	21 (9)	34 (14)		139 (68)	32 (16)	34 (16)
Highest educational level (father) 2003	1171				1026				1025				847			
1 (> 13 yr)		276 (92)	19 (6)	5 (2)		215 (85)	16 (6)	23 (9)		224 (83)	26 (10)	20 (7)		194 (81)	31 (13)	15 (6)
2 (10–13 yr)		496 (84)	54 (9)	41 (7)		384 (74)	58 (11)	77 (15)		418 (83)	52 (10)	37 (7)		284 (72)	62 (16)	50 (12)
3 (< 10 yr)		235 (84)	24 (9)	21 (7)		172 (68)	40 (16)	41 (16)		195 (78)	24 (10)	29 (12)		138 (65)	39 (19)	34 (16)
Family functioning^c^	1088				967				932				773			
Good		728 (87)	63 (8)	44 (5)		567 (78)	76 (10)	87 (12)		573 (81)	71 (10)	63 (9)		419 (71)	91 (16)	79 (13)
Poor		208 (82)	29 (12)	16 (6)		158 (67)	30 (12)	49 (21)		186 (83)	26 (11)	13 (6)		136 (74)	29 (16)	19 (10)

^a^Yearly household income categorized at 33.3rd;66.6th percentile

^b^Labour market participation dichotomized ≥0.80 (high score)

^c^Family functioning, measured at age 15

A higher proportion of overweight and obesity at age 18 and 21 was observed in both genders if they grew up with lower educated parents or if their mothers had a low LMP during their early childhood. Among boys, a higher proportion of obesity at age 18 and 21 was also observed in low income families or if they had a father with low LMP during their early childhood.

A higher proportion of overweight and obesity at age 18 and 21 was likewise observed in both genders, if they grew up with lower educated parents during their late childhood. A higher proportion of obesity was observed among both genders at age 21 in lower income families during their late childhood. Furthermore, among girls, a higher proportion of overweight and obesity at age 18 and 21 was observed if their parent’s had low LMP or the girls reported poor “family functioning” during their later childhood.

15-year-old girls had increased risk of overweight, if they reported poor “family functioning”; OR = 1.7, 95% CI 1.1;2.7 (table not shown).

15-year-old boys had an increased risk of overweight, if their fathers had a low LMP or the boys came from families with parents having a low level of education during their early childhood, with estimates ranging from OR = 1.6, 95% CI 0.9;2.9 to OR = 2.2, 95% CI 1.2;3.8. Parental low educational level during their late childhood almost doubled boys’ risk of overweight at 15 years of age, OR = 1.9, 95% CI 1.1;3.3. It also gave the impression that boys who grew up in low income families had some increased risk of overweight at age 15, although this being imprecise results (OR = 1.7, 95% CI 0.9;3.1) (table not shown).

Girls, whose mother had a lower educational level in their early childhood, had increased risk of overweight and obesity at age 21, RR = 1.9, 95% CI 1.0;3.8 and RR = 2.1, 95% CI 1.1;3.9, respectively. This tendency was also seen with father’s lower educational level, which increased the risk of overweight and obesity at both age 18 and 21, with estimates ranging from RR = 1.8, 95% CI 1.0;3.4 to RR = 5.2, 95% CI 1.4;19.3. Likewise, reporting poor “family functioning” increased girls’ risk of overweight at the age of 18 and obesity at the age of 21 between 1.6 and 2 times (Table 2).

**Table 2:** The association between yearly household income (income), labour market participation (LMP), parental highest educational level (highest edu) and family functioning (family func) in early childhood and overweight and obesity at age 18 or 21

Early Childhood	Girls												Boys											
	18						21						18						21					
	n	RR		n	ARR^d^ (95% CI)		n	RR		n	ARR^d^ (95% CI)		n	RR		n	ARR^d^ (95% CI)		n	RR		n	ARR^d^ (95% CI)	
		Overweight	Obesity		Overweight	Obesity		Overweight	Obesity		Overweight	Obesity		Overweight	Obesity		Overweight	Obesity		Overweight	Obesity		Overweight	Obesity
Income^a^	1200			970			1060			864			1030			840			861			698		
High (ref.grp.)		1	1		1	1		1	1		1	1		1	1		1	1		1	1		1	1
Medium		1.6	1.5		1.5 (0.9;2.6)	1.4 (0.7;2.9)		1.2	1.6		0.7 (0.4;1.2)	1.4 (0.9;2.4)		0.8	1.1		0.7 (0.4;1.1)	1.0 (0.5;1.9)		0.9	1.5		0.7 (0.4;1.1)	1.1 (0.6;2.0)
Low		1.3	1.1		0.9 (0.5;1.9)	0.9 (0.4;2.1)		1.0	1.5		0.5 (0.3;1.0)	1.1 (0.6;1.9)		1.1	1.8		0.7 (0.3;1.4)	1.2 (0.6;2.4)		0.8	1.8		0.5 (0.3;1.0)	0.9 (0.5;1.9)
LMP^b^ (mother)	1239			970			1085			864			1066			840			883			698		
High score (ref.grp.)		1	1		1	1		1	1		1	1		1	1		1	1		1	1		1	1
Low score		1.6	1.2		1.3 (0.8;2.1)	1.0 (0.6;2.0)		1.7	1.3		1.6 (1.0;2.6)	1.2 (0.8;1.8)		1.1	1.5		1.1 (0.6;2.0)	1.3 (0.7;2.3)		1.8	2.3		2.0 (1.2;3.2)	2.2 (1.3;3.8)
LMP^b^ (father)	1228			970			1074			864			1062			840			881			698		
High score (ref.grp.)		1	1		1	1		1	1		1	1		1	1		1	1		1	1		1	1
Low score		1.3	0.7		1.4 (0.7;2.9)	0.8 (0.3;2.2)		1.0	0.9		0.7 (0.3;1.5)	0.8 (0.4;1.6)		1.4	2.0		1.1 (0.4;2.8)	2.8 (1.3;6.1)		0.7	1.4		0.7 (0.3;2.1)	1.6 (0.7;4.1)
Highest edu^e^ (mother)1989	1165			970			1034			864			1001			840			841			698		
1 (> 13 yr) (ref.grp.)		1	1		1	1		1	1		1	1		1	1		1	1		1	1		1	1
2 (10–13 yr)		1.2	1.2		0.8 (0.4;1.6)	0.7 (0.3;1.7)		1.4	1.3		0.9 (0.5;1.7)	1.0 (0.5;1.7)		1.4	1.0		1.4 (0.7;2.5)	0.8 (0.4;1.6)		1.8	1.3		1.7 (1.0;3.0)	0.7 (0.4;1.4)
3 (< 10 yr)		1.9	2.9		1.4 (0.7;2.7)	1.9 (0.8;4.7)		3.2	2.8		1.9 (1.0;3.8)	2.1 (1.1;3.9)		1.5	2.4		1.4 (0.7;2.8)	1.5 (0.7;3.2)		1.7	2.4		1.4 (0.7;2.7)	1.3 (0.7;2.6)
Highest edu^e^ (father) 1989	1119			970			984			864			965			840			801			698		
1 (> 13 yr) (ref.grp.)		1	1		1	1		1	1		1	1		1	1		1	1		1	1		1	1
2 (10–13 yr)		1.7	4.4		2.1 (1.0;4.3)	4.5 (1.3;15.7)		2.3	1.9		2.5 (1.2;5.1)	1.8 (1.0;3.4)		1.2	1.2		1.1 (0.6;1.9)	1.0 (0.5;2.0)		1.5	2.2		1.6 (0.9;2.8)	1.9 (0.9;4.0)
3 (< 10 yr)		1.6	4.9		1.4 (0.6;3.2)	5.2 (1.4;19.3)		3.4	2.2		2.6 (1.2;5.8)	1.8 (0.9;3.6)		1.3	2.1		1.1 (0.5;2.2)	1.6 (0.8;3.5)		1.8	3.0		1.8 (0.9;3.4)	2.4 (1.1;5.4)
Family func^c^	1088			970			967			864			932			840			773			698		
Good (ref.grp.)		1	1		1	1		1	1		1	1		1	1		1	1		1	1		1	1
Poor		1.6	1.3		1.6 (1.0;2.7)	1.2 (0.6;2.3)		1.4	2.0		1.5 (0.9;2.5)	2.0 (1.3;3.1)		1.1	0.6		1.1 (0.6;1.9)	0.5 (0.3;1.0)		1.0	0.7		0.9 (0.5;1.5)	0.5 (0.3;1.0)

^a^Yearly household income categorized at 33.3rd and 66.6th percentile

^b^Labour market participation dichotomized ≥0.80 (high score)

^c^Family functioning, measured at age 15

^d^Mutual adjusted for other exposure variables, birthweight and split home 1989

^e^Highest educational level

Boys, whose mother had low LMP during their early childhood, had about twice the risk of overweight and obesity at the age of 21 (RR = 2.0, 95% CI 1.2;3.2, RR = 2.2, 95% CI 1.3;3.8). Boys, whose father had a lower level of education during their early childhood, had up to 2.4 times increased risk of obesity at age 21 (RR = 2.4, 95% CI 1.1;5.4). Furthermore, father’s low LMP during early childhood increased boys’ risk of obesity at the age of 18, RR = 2.8, 95% CI (1.3;6.11) (Table 2).

Girls, whose mother had a low level of education during their late childhood, had between 2 and 2.2 times increased risk of obesity at age 18 and 21. Father’s lower level of education in late childhood almost increased girls’ risk of obesity 4-fold at age 18 (RR = 3.7, 95% CI 1.2;11.9) and more than doubled the risk of overweight at age 21 (RR = 2.5, 95% CI 1.2;5.2). Reporting poor “family functioning” also increased girls’ risk of obesity at age 21, RR = 1.7, 95% CI 1.1;2.7. It seemed that girls who grew up in lower income families or experienced their parent’s having low LMP during their later childhood had increased risk of obesity at age 18 and 21 though the estimates were inaccurate (Table 3).

**Table 3:** The association between yearly household income (income), parental labour market participation (LMP), parental highest educational level (highest edu) and family functioning in late childhood and overweight or obesity at age 18 or 21

Late Childhood	Girls												Boys											
	18						21						18						21					
	n	RR		n	ARR^f^ (95% CI)		n	RR		n	ARR^f^ (95% CI)		n	RR		n	ARR^f^ (95% CI)		n	RR		n	ARR^f^ (95% CI)	
		Overweight	Obesity		Overweight	Obesity		Overweight	Obesity		Overweight	Obesity		Overweight	Obesity		Overweight	Obesity		Overweight	Obesity		Overweight	Obesity
Income^a^	1222			970			1072			862			1053			837			876			697		
High (ref.grp.)		1	1		1^g^	1^g^		1	1		1^g^	1^g^		1	1		1^g^	1^g^		1	1		1^g^	1^g^
Medium		1.2	1.8		0.8 (0.4;1.4)	2.1 (0.9;5.0)		0.8	1.6		0.6 (0.4;1.2)	1.3 (0.7;2.3)		0.8	1.4		0.9 (0.5;1.6)	1.5 (0.7;3.0)		0.8	1.2		0.8 (0.4;1.3)	0.8 (0.4;1.5)
Low		1.4	1.3		0.7 (0.3;1.5)	2.0 (0.7;5.9)		1.1	1.8		1.0 (0.5;2.0)	1.8 (0.9;3.5)		1.1	1.6		0.9 (0.4;2.0)	1.3 (0.5;3.3)		0.6	1.4		0.6 (0.3;1.4)	1.0 (0.4;2.2)
LMP^b^ (mother)	1228			972			1076			862			1059			838			879			698		
High score (ref.grp.)		1	1		1^h^	1^h^		1	1		1^h^	1^h^		1	1		1^h^	1^h^		1	1		1^h^	1^h^
Low score		1.8	1.8		1.6 (0.9;2.8)	1.6 (0.8;3.2)		1.5	1.4		1.1 (0.6;2.0)	1.3 (0.8;2.1)		1.1	1.2		1.1 (0.6;2.1)	0.6 (0.3;1.3)		0.9	1.1		1.1 (0.6;2.0)	0.9 (0.5;1.8)
LMP^b^ (father)	1213			972			1057			862			1049			838			868			698		
High score (ref.grp.)		1	1		1^i^	1^i^		1	1		1^i^	1^i^		1	1		1^i^	1^i^		1	1		1^i^	1^i^
Low score		1.1	1.3		1.3 (0.6;2.8)	2.0 (0.8;5.1)		1.6	1.7		1.5 (0.7;3.2)	1.6 (0.8;3.1)		0.9	1.6		0.6 (0.2:1.9)	1.0 (0.4;2.8)		0.2	0.9		0.1 (0.0;0.9)	0.7 (0.3;2.2)
Highest edu^d^ (mother) 2003	1225			972			1076			862			1048			838			876			698		
1 (> 13 yr) (ref.grp.)		1	1		1	1		1	1		1	1		1	1		1	1		1	1		1	1
2 (10–13 yr)		1.5	1.5		1.2 (0.7;2.2)	1.1 (0.5;2.4)		1.6	1.7		1.3 (0.7;2.3)	1.4 (0.8;2.3)		1.3	0.9		1.3 (0.8;2.2)	0.9 (0.5;1.6)		1.6	1.5		1.6 (1.0;2.7)	1.0 (0.6;1.8)
3 (< 10 yr)		2.1	3.3		1.8 (0.9;3.5)	2.2 (0.9;5.2)		2.9	2.8		1.8 (0.9;3.4)	2.0 (1.1;3.7)		1.1	2.3		0.9 (0.4;1.8)	1.8 (0.9;3.6)		1.5	2.2		1.6 (0.9;3.0)	1.6 (0.9;3.1)
Highest edu^d^ (father) 2003	1171			972			1026			862			1025			838			847			698		
1 (> 13 yr) (ref.grp.)		1	1		1	1		1	1		1	1		1	1		1	1		1	1		1	1
2 (10–13 yr)		1.6	4.6		1.5 (0.8;2.8)	3.1 (1.0;9.3)		2.0	1.9		2.1 (1.1;4.0)	1.4 (0.8;2.4)		1.1	1.0		1.2 (0.7;2.2)	0.9 (0.4;1.7)		1.4	2.3		1.5 (0.9;2.6)	2.5 (1.3;5.0)
3 (< 10 yr)		1.5	4.9		1.1 (0.5;2.4)	3.7 (1.2;11.9)		3.1	2.2		2.5 (1.2;5.2)	1.5 (0.8;2.9)		1.1	1.7		1.2 (0.6;2.4)	1.3 (0.6;2.8)		1.8	3.2		1.9 (1.0;3.6)	2.9 (1.4;6.4)
Family Func^c^	1088			972			967			862			932			838			773			698		
Good (ref.grp.)		1	1		1	1		1	1		1	1		1	1		1	1		1	1		1	1
Poor		1.6	1.3		1.5 (0.9;2.5)	1.0 (0.5;2.0)		1.4	2.0		1.3 (0.8;2.1)	1.7 (1.1;2.7)		1.1	0.6		1.1 (0.6;1.9)	0.5 (0.3;1.1)		1.0	0.7		1.1 (0.7;1.8)	0.6 (0.3;1.2)

^a^Yearly household income categorized at 33.3rd and 66.6th percentile

^b^Labour market participation dichotomized ≥0.80 (high score)

^c^Family functioning, measured at age 15

^d^Highest educational level

^f^Mutual adjusted for other exposure variables, birth-weight and split home 2003

^g^Mutual adjusted for other exposure variables, birth-weight, split home 2003 and early childhood income

^h^Mutual adjusted for other exposure variables, birth-weight, split home 2003 and mother’s LMP in early childhood

^i^Mutual adjusted for other exposure variables, birth-weight, split home 2003 and father’s LMP in early childhood

Boys, whose mother had a lower level of education during their late childhood, appeared to have some increased risk of obesity at the ages of 18 and 21, although the estimates were imprecise; (RR = 1.8, 95% CI 0.9;3.6, RR = 1.6, 95% CI 0.9;3.1). Father’s lower level of education in late childhood almost tripled boys’ risk of overweight and obesity at age 21, RR = 1.9, 95% CI 1.0;3.6, RR = 2.9, 95% CI 1.4;6.4, respectively (Table 3).

## Discussion

This study showed that growing up in families with parent’s having a low level of education in early or late childhood increased the risk of overweight and obesity at age 18 and 21 in both genders, where especially father’s low level of education appeared to be a quite strong risk factor in both genders, despite somewhat wide confidence intervals. We also found that girls, who reported poor “family functioning” in early or late childhood had increased risk of overweight and obesity at age 18 and 21, which was not seen among boys. Among boys, results showed that growing up in families with parent’s having low LMP during early childhood increased their risk of overweight and obesity at age 18 and 21.

To our knowledge, this is the first study to examine how childhood SEP relates to overweight and obesity, using a longitudinal study-design with 14 years of register-based exposure information. Furthermore, this was supplemented with the social factor “family functioning” to facilitate the subjective perception of childhood social conditions on the family level, which is not captured by the objective SEP measures.

Our results were in line with findings from the study by Kestila et al., who examined the association between childhood social circumstances and overweight and obesity in early adulthood in a cross-sectional design. The authors found a strong inverse association between parental educational level and obesity in both genders [34]. These results are also supported by Mathiessen et al., who found that educational level of the parents was inversely associated with their off-spring being overweight [35]. Morgen et al. found that 14–16-year-old girls of lower parental SEP had more than four times the risk of developing overweight/obesity at age 21, compared to girls of higher parental SEP [36]. In our study, we found that parental lower educational level in early or late childhood may increase the risk of overweight and obesity at age 18 and 21 up between 1.8 and 3-fold among both genders.

Al-Emranie et al. examined the association between five-year weight gain among adults and SEP in childhood and adulthood. They found a significant association between childhood SEP and obesity among males aged 29–39, thereby suggesting that the socioeconomic gradient is even more prominent in relation to obesity [37]. Results from our study showed that parental low LMP in early childhood was associated with increased risk of overweight and obesity in primarily boys, with a more than 2-fold increased risk of obesity at the age of 18 and 21.

Bann et al. examined how childhood and adult SEP relates to BMI across adulthood in three national British birth cohorts. They found that father’s occupational class at age 10/11 was associated with higher adult BMI in both genders [38]. These findings are partly supported by results from our study concerning girls showing that low parental LMP in late childhood was associated with increased risk of overweight and obesity at age 18, although the findings were inaccurate. Among boys our results indicated that parental LMP in late childhood may be less important for boys’ risk of later overweight and obesity.

Overall, our findings indicate that childhood low SEP at the family level is associated with increased risk of overweight and obesity in adolescence and early adulthood. As mentioned in the background, a recent theory suggests that obesity may be a healthy active response to a future lack of energy caused by the sense of e.g. social insecurity in the family [8]. This could be a plausible explanation for a possible pathway between low childhood SEP and the development of obesity in a well-fare society with easy accessibility to rich calories-dense food. We did not find strong associations between low household income in childhood and later overweight/obesity, which may be due to this population living in a well-fare society, where a family may have a reasonable living despite a rather low income. However, we saw a tendency towards an increased risk of obesity at age 18 and 21 among girls, who grew up in low income families in late childhood, when the associations were adjusted for the early childhood income indicating that the timing of this exposure may be relevant among girls, but not boys.

Parental lower educational level(s) during early and late childhood were quite consistent risk factors for overweight and obesity in both genders in this youth cohort. Parental low LMP in early childhood was primarily a risk factor for boys, and for girls there was a tendency in late childhood to influence girls’ future risk of overweight and obesity. Parental lower educational level and parental low LMP may negatively affect the psychosocial security experienced in families due to e.g. job insecurity, living in poorer residential area and perhaps also an unhealthy life style, which may affect the children. Due to role modeling, children reflect themselves in their parents, so when boys experience their father having low LMP during early childhood, this may increase boys’ feeling of perceived social insecurity in daily life, which may be translated into psychological processes with possible future biological consequences [8]. Lower educated parent’s and parent’s with low LMP are perhaps also more likely to pass on poorer eating habits to the children [39], which combined with increasing sedentary behavior and risk behavior may tract into adolescence and adulthood and thereby also contribute to an enhanced risk of overweight and obesity.

A recent review conducted primarily on cross sectional studies concluded, that poor “family functioning” was associated with increased risk of overweight and obesity among children and adolescent aged 3–17 [15]. We observed gender differences in our study, where reporting poor “family functioning” at age 15 was a risk factor for overweight and obesity in adolescence and early adulthood in girls, but not among boys. Perhaps weight-gaining in boys during adolescence and early adulthood are less affected by how the nearest family function, compared to girls due to e.g. different coping strategies or life styles [40].

This cohort study had several strengths. The initial study response rate was 78%, which somewhat declined at the latter rounds. The study covered up to 21 years of follow up and used register-based information to define most of the exposure variables, which resulted in few missing values. The exposure variable “family functioning” was applied to uncover the child’s experiences of the social conditions in the family during childhood. By adding the subjective perspective in terms of this social factor, we emphasize the importance of this influence on the physical health of young people.

The prospective design is suitable to observe potential changes over time and we consider this to be an appropriate way of studying this kind of associations. Also, applying register-based exposure variables diminishes the risk of differential information bias on these variables.

Participating in surveys may be prone to selection bias; that is if non-participation is associated with both exposures and outcomes. In this cohort, we found non-participants to be significantly different from participants with respect to the exposure variables, however, we do not have any information on height and weight from the non-participants, so it is not possible to disentangle whether any selection was differential. Non-participation and drop-outs in the same cohort was examined in a previous study by Winding et al. and results showed that neither non-participation nor drop-outs influenced significantly on the size of the measured associations [19].

The main limitation of the study was that the outcome was based on self-reported height and weight and consequently prone to misclassification. Participants, who are overweight, are probably more likely to underestimate their weight [41], which may be most pronounced in girls [42]. This increases the risk of underestimating the associations between the exposures and the outcome and hence bias towards the null-hypothesis. We believe that due to the study design the risk of differential misclassification of the outcome was small. We applied the self-reported variable “family functioning” along with the outcome reported from the baseline questionnaire at age 15 and we are aware that these findings are cross-sectional and cannot tell us anything about causality.

We decided to imply the additional cut-offs for obesity from the Global database on BMI due to a relatively low prevalence of obese participants in this cohort according to conventional World Health Organization-guidelines. We believe that applying the additional cut-off seems reasonable in this young healthy population.

All the associations in the study were mutually adjusted for the other exposure variables, but these adjustments did not alter the results much. We did not find strong correlations between e.g. household income and highest educational level in this study. This may be explained by the fact that household income in Denmark not necessarily reflects a person’s level of education. An unskilled worker in a factory often earns a rather high salary compared to e.g. health care workers with a short or medium long education. For the early childhood adjustment’s we applied split home 1989, however we repeated the analyses with split home 1991 instead, because the first couple of years after the birth of a child may be a difficult time for the parents’ relationship and one could suspect that more families may split up during these years. Applying split home 1991 did not change the estimates.

A previous examination of the study setting concluded that the participants of this youth cohort are comparable to young people in other parts of Denmark [43]. Therefore, the results of this study may be transferred to young people with similar environmental and social conditions to this Danish cohort, when taking the above-mentioned limitations into account.

## Conclusion

In this study, we found that parental lower educational level during childhood was associated with an increased risk of overweight and obesity in adolescence and early adulthood in both genders. Father’s lower educational levels during early or late childhood were the strongest risk factors for overweight and obesity at age 18 and 21 with as much as fivefold increased risks.

Parental low LMP during early childhood was a risk factor for overweight and obesity at age 18 and 21 in primarily boys, where reporting poor “family functioning” was a risk factor for overweight and obesity in girls only. The timing of SEP in childhood appears to be gender-specific according to some of the parental socioeconomic exposure variables; girls seems to be primarily influenced by the later childhood lower income and parent’s low LMP, where it appeared to be parent’s low LMP in the earlier part of the childhood which may influence boys’ risk of future overweight and obesity the most. The results should, however, be interpreted with caution due to imprecise estimates with wide confidence intervals.

Lower SEP in childhood is associated with overweight and obesity in adolescence and early adulthood in Denmark despite this being a well-fare society, where rules and regulations aim to reduce inequality. Further research is required to disentangle some of the underlying mechanisms and to be able to target relevant support to prevent overweight and obesity related to childhood conditions.

## Additional file

Additional file 1: **Table S1.** Spearman’s rank correlation matrix of exposure variables in early (0–8 years) and late childhood (9–14 years). (DOCX 30 kb)

## Abbreviations

ARR: Adjusted Relative Risks
BMI: Body Mass Index
CI: Confidence interval
CPR: Civil Registration Number
E.g.: Exempli gratia
LMP: Labour market participation
SEP: Socioeconomic position
Yr: Year
