# Supplementary material for: How does childhood socioeconomic position affect overweight and obesity in adolescence and early adulthood: a longitudinal study
Source: BMC Obes. 2018 Dec 3;5:34. doi: 10.1186/s40608-018-0210-8 (PMC6276194; doi:10.1186/s40608-018-0210-8)
Supplement: Supplementary file 1 [file 40608_2018_210_MOESM1_ESM.docx]

Table S1. Spearman´s rank correlation matrix of exposure variables in early (0-8 years) and late childhood (9-14 years)

	yr08mean	yr9_14_mean	highEdu_m89	highEdu_m03	highEdu_f89	highEdu_f03	yr08wps_m	yr9_14wps_m	yr08wps_f	yr9_14wps_f	yr15famfunc	splithome_89	splithome_03
yr08mean	1.0000												
yr9_14_mean	0.7352	1.0000											
highEdu_m89	0.3090	0.2970	1.0000										
highEdu_m03	0.2294	0.2524	0.8503	1.0000									
highEdu_f89	0.3197	0.3307	0.3437	0.3285	1.0000								
highEdu_f03	0.2911	0.3238	0.3373	0.3327	0.9414	1.0000							
yr08wps_m	0.3298	0.2834	0.2920	0.2098	0.1713	0.1519	1.0000						
yr9_14wps_m	0.2803	0.2950	0.2973	0.2311	0.1479	0.1452	0.4689	1.0000					
yr08wps_f	0.3611	0.2829	0.1431	0.1190	0.2073	0.1687	0.1903	0.1881	1.0000				
yr9_14wps_f	0.2593	0.2733	0.1292	0.1055	0.1909	0.1779	0.1492	0.1920	0.4792	1.0000			
yr15famfunc	-0.0666	-0.0943	-0.0724	-0.0733	-0.0779	-0.0772	-0.0543	-0.0429	-0.1027	-0.0792	1.0000		
splithome_89	0.0009	-0.0083	0.0311	0.0458	0.0057	0.0013	-0.0088	-0.0228	0.0123	0.0026	-0.0164	1.0000	
splithome_03	-0.2727	-0.3655	-0.0934	-0.0641	-0.1290	-0.1366	-0.1255	-0.1763	-0.1573	-0.1720	0.1138	0.0351	1.0000

yr08mean: mean yearly household income in early childhood (0-8 years)
yr9_14_mean: mean yearly household income in late childhood (9-14 years)
highEdu_m89: mother´s highest educational level in 1989
highEdu_m03: mother´s highest educational level in 2003
highEdu_f89: father´s highest educational level in 1989
highEdu_f03: father´s highest educational level in 2003
yr08wps_m: mean Labour market participation in early childhood (0-8 years)
yr9_14wps_m: mean Labour market participation in late childhood (9-14 years)
yr08wps_f: mean Labour market participation in early childhood (0-8 years)
yr9_14wps_f: mean Labour market participation in late childhood (9-14 years)
yr15famfunc: mean family fuctioning age 15
splithome_89: living with both parents or not in 1989
